# Therefore Its Name Is Called Babel …

**DOI:** 10.1177/2041669515593654

**Published:** 2015-08-19

**Authors:** Jan Koenderink

But the Lord came down to see … the tower which the sons of men had built. And the Lord said, “Indeed the people are one and they all have one language, and this is what they begin to do; … Come, let Us go down and there confuse their language, that they may not understand one another’s speech.” … Therefore its name is called Babel, because there the Lord confused the language of all the earth; … (King James Bible, Genesis 11)

**Figure 1.**
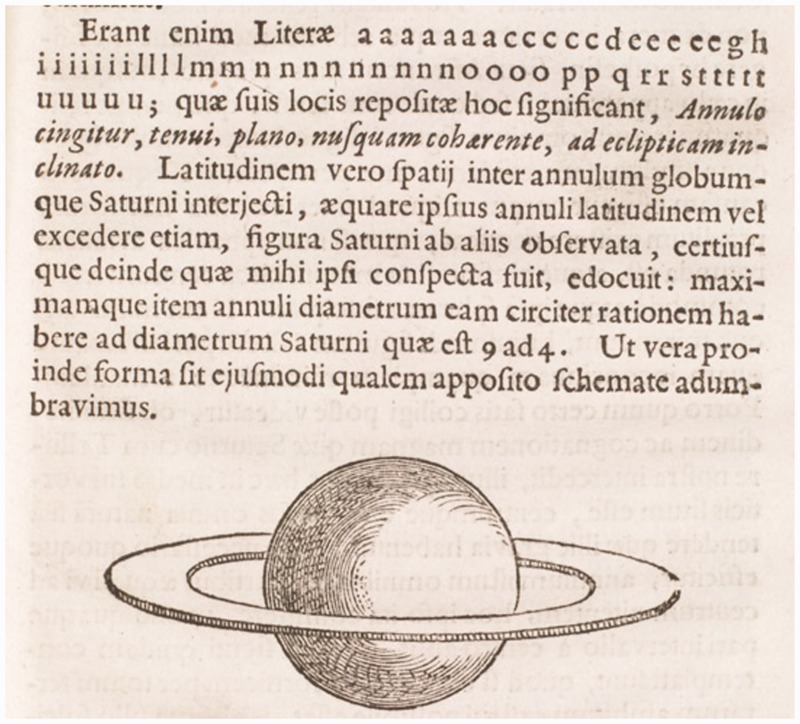
The drawing evidently illustrates a 3D object in reflective thought, rather than a record of immediate visual awareness.

**Figure 2.**
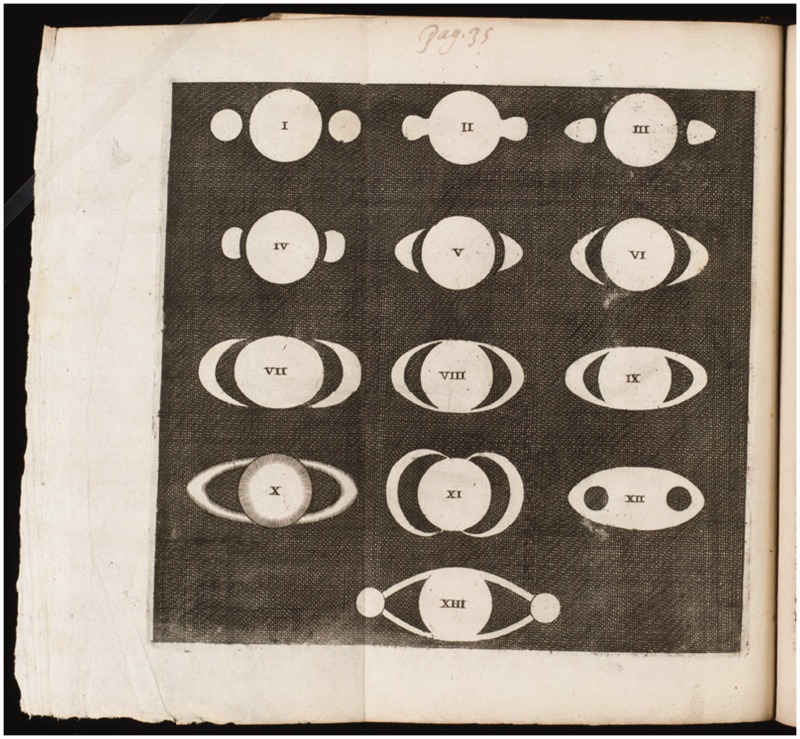
What the others saw: “handlesrdquo;, a pair of “moons”, some kind of “aura” or “halo”. I is by Galileo from 1610; latests date from 1650. All are 2D patterns, apparently these observers reported on their immediate visual awareness. IV–VII are theoretical suggestions by Hevelius, also 2D.

In 1656, Christiaan Huygens enriched the world with the remarkable publication “aaaaaaacccccdeeeeeghiiiiiiillllmmnnnnnnnnnooooppqrrstttttuuuuu” in a little booklet *De Saturni luna observatio nova* about his discovery of a moon of Saturn. The anagram made little impact, but those in the know understood that he had made a remarkable discovery. A few years later, Huygens came out with his *Systema Saturnium, sive de causis mirandorum Saturni phaenomenon, et comite ejus planeta novo* (The Hague: Adrian Vlacq, 1659) that contained the solution: “Annulo cingitur, tenui, plano, nusquam cohaerente, ad eclipticam inclinato.” Translated into modern English: “It is surrounded by a thin flat ring, nowhere touching, and inclined to the ecliptic” (Figure 1). This solved the enigma of the *ansea* (English: handles) and soon astronomers reported to “see” a ring instead. People had seen the handles as if they were looking at the picture of a jug in a children’s coloring book (Figure 2), whereas Huygens saw a three-dimensional object. It was a problem in applied perception.

There are several things to learn from this. An anagram is a puzzle with numerous potential “solutions.” Translation is impossible. Decyphering a code is not translation either. It will take you less than a minute to crack this childish code: *nehcuar thcin*, but then you are still left to do a translation. Translation as decoding applied to “the spirit is willing, but the flesh is weak” to Russian and back reputedly yielded “the vodka is strong but the steak is lousy” (John Hutchins, MT News International 11 [June 1995], 17–18).

Translation proper implies *understanding the original* and expressing its meaning in another language. My favorite book on this problem is George Steiner’s *After Babel* (1975, Oxford University Press). It should be required reading for translators. A word-for-word “translation” ignores meaning and is often impossible anyway. This not infrequently leads to serious misunderstandings. For instance, the fact that the German *Wirklichkeit* and *Realität* both translate to “reality” in English is at least partly behind the chasm between “continental” and “analytic” philosophy. Jakob von Uexküll’s *Merkton* is usually rendered as *affordance*. Indeed, James Gibson probably got the idea by way of German psychologists who moved to the United States during the 1930s. But Gibson finds “throwability” in a stone, whereas von Uexküll finds it in visual awareness: Thus, “literal translation” seriously misrepresents the meaning. Such misfits not only apply to languages but also to times. For instance, I meet students who are puzzled when they notice that I apparently differentiate between “mind” and “brain.” It shows my age.

Latin used to be the language of science. Huygens was fluent as a child, some education! Till the mid 20th century scientific papers came in German, French, English, Italian, Russian, and various other languages (no particular order intended). A notable attempt to switch to Esperanto in the early 20th century misfired (Wilhelm Ostwald, “Die Organisierung der Organisatoren durch die Brücke”, München: Brücke, 1912). After World War II, English has taken over for all practical purposes. Consequently, many students are unable to read the bulk of the literature published earlier, with the unfortunate result that the wheel is frequently reinvented and credits go to inappropriate places.

Here’s the good news: *i-Perception* is going to help! The board has the intention to consider contributions that focus on translations of our “roots.” We have been fortunate enough to be able to publish two of such contributions in this issue. They are of a slightly different character and may be taken to set the intended tone.

More formally, here is an account of our intentions. The overall rule is that the contributions should be of genuine interest to readers of *i-Perception*. This leads to various considerations that might help you focus. I’m sure there are more, so don’t feel pinned down by rules. If you have plans, it is perhaps wise to discuss them with us first. Here is the list of general considerations (for details of the submission procedure and instructions for authors, see http://submission.perceptionweb.com/supplement/instructions/ip/authors.html):

– aim for the current appreciation of some forgotten gem, don’t translate for translation’s sake;
– whereas a short paper might merit translation *in toto*, it will often be preferable to select vital parts and summarize the remainder;
– because of the wide ranging interest and expertise of the readership, it will almost always be preferable to augment the translation with some text that puts the work in perspective and relates it to modern day problems of the field.
– translations may be of historical sources, but remember that there are plenty of recent publications in Japanese, Swedish, and so forth that are a closed book to *i-Perception*’s readership!
– if translations of a work are already available, that may or may not mean, that a novel attempt would be useless! Because translation is about conveying meaning, translations should reflect modern understanding too.

This is evidently an experiment. If you have ideas or proposals we’re ready to listen.

